# Nasal Lymphoma Presenting With Contralateral Proptosis and Vision Changes: A Case Report and Literature Review

**DOI:** 10.7759/cureus.11287

**Published:** 2020-11-01

**Authors:** Hugh J Kim, Laura M Kim, Brian W Rotenberg

**Affiliations:** 1 Otolaryngology, Western University, London, CAN

**Keywords:** non-hodgkin lymphoma, nasal mass, proptosis, vision changes, cranial neuropathies, contralateral symptoms, case report, literature review, diagnostic challenge

## Abstract

Non-Hodgkin lymphoma (NHL) is a hematological malignancy that can sometimes originate from the nasal cavities and paranasal sinuses. Patients who present with these tumors typically report sinonasal symptoms. However, a diagnostic challenge can arise when a patient’s primary complaints include unique complex symptoms limited to the contralateral side of the tumor. This report describes the case of an 83-year-old man who presented to our center with a left-sided mass and right-sided proptosis with vision loss. After a nasal biopsy was taken, the patient was referred to the ophthalmology department to diagnose the cause of his ocular symptoms, which were not believed to be related to the mass. When biopsy results later returned as diffuse large B-cell lymphoma (DLBCL), an emergent repeat biopsy following lymphoma protocol was performed to confirm the diagnosis. A CT scan of the head and orbits showed generalized enlargement of the right optic nerve and extraocular muscles, and a positron emission tomography (PET) scan showed increased 18F-fluorodeoxyglucose (FDG) uptake in the right ethmoid sinus and orbit. The left-sided mass and right-sided symptoms resolved simultaneously with chemotherapy. This is the first documented case of a sinonasal mass causing ocular symptoms exclusively on the contralateral side. The presented diagnostic challenge highlights the importance of thorough investigations.

## Introduction

Non-Hodgkin lymphoma (NHL) is a hematological malignancy accounting for 4.6% of all invasive cancers in the United States [1]. NHL is a disease of the lymphoreticular system, but 30% may originate from extra-nodal sites [2], such as the liver, soft tissue, dura, bone, stomach, intestine, and bone marrow [3]. In the Western world, primary NHL of the nasal cavities and paranasal sinuses are rare, accounting for 1.6% of all NHL, and are most commonly of the large B-cell subtype [4]. They often present with symptoms of nasal obstruction, nasal discharge, and epistaxis [5]. On the other hand, there are cases of sinonasal NHL that can present with atypical features. For example, ocular symptoms such as vision loss and proptosis may be the initial presenting symptoms with ethmoid and sphenoid sinus involvement [6].

Proptosis, defined as an abnormal forward displacement of the eye, is a nonspecific symptom. Therefore, the evaluation of unilateral proptosis in an adult patient should include a broad differential diagnosis that considers traumatic, endocrine, inflammatory, vascular, and neoplastic processes. A thorough multi-disciplinary workup with medical history, physical examination, and imaging investigations is warranted to identify the correct disease process [7]. The otolaryngologist has expertise in recognizing proptosis arising secondary to mass lesions in the nose and paranasal sinuses. Classically, this occurs ipsilaterally due to direct invasion or displacement of the orbit by the disease [8]. To our knowledge, proptosis in association with sinonasal masses on the contralateral side has been neither reported nor explained.

In this report, we discuss an atypical case of diffuse large B-cell lymphoma (DLBCL) in the nasal cavity with associated proptosis of the contralateral eye that presented a diagnostic challenge.

## Case presentation

An 83-year-old man was referred to our outpatient otolaryngology clinic due to findings of a left nasal mass on CT and MRI. He had a three-month history of left-sided nasal obstruction and right-sided proptosis accompanied by intermittent diplopia and severe right-sided headache. One month after the onset of symptoms, he also experienced right-sided facial nerve weakness with sialorrhea and pain in the temporomandibular joint with mastication. Furthermore, there had been symptoms of mild right-sided facial congestion and hyposmia. He denied any facial pain or nasal discharge. All the symptoms excluding the nasal obstruction were limited to the right-hand side.

Upon general inspection, the patient appeared well and in no apparent distress. Testing of cranial nerves II to XII revealed mild V2 distribution numbness and lower facial droop on the right-hand side. There was also absent abduction with esotropia of the right eye. The remaining cranial nerve exam findings were within normal limits. Flexible fiber-optic nasopharyngoscopy confirmed the presence of a left-sided nasal mass. The mass appeared polypoid in nature and was significantly narrowing his left nasal cavity. A biopsy was taken under local anesthetic and functional endoscopic sinus surgery was planned for symptomatic management.

**Figure 1:**
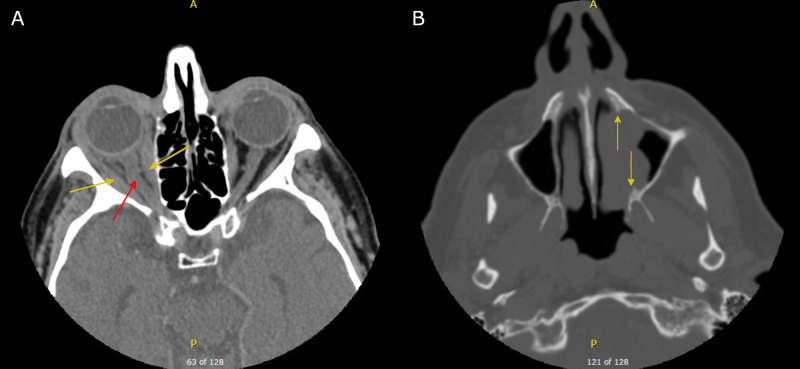
Non-contrast axial CT of the head and orbits. A) Soft tissue window showing diffuse enlargement of right extraocular muscles (yellow arrows) and tortuous right optic nerve (red arrow). B) Bone window showing a left-sided nasal tumor extending into the left maxillary sinus, with yellow arrows marking the extent of bone erosion. The coronal and sagittal reformats had incomplete visualization of the tumor vertically and are not shown.

At this point, it was not believed that this left-sided mass was the cause of his right-sided symptoms, and he was referred to our ophthalmology colleagues for a diagnosis. They ordered a CT scan, which showed diffuse right orbital abnormality involving optic nerve thickening, generalized enlargement of the right extraocular muscle (EOM), and infiltration of the orbital fat (Figure 1A). The left nasal mass was seen extending into the left maxillary sinus with demineralization of the medial wall (Figure 1B).

The results of the in-office biopsy returned as DLBCL. This was out of keeping with our clinical impression. Indeed, our patient appeared well, without B symptoms or hematological abnormalities on blood work. To obtain more tissue for confirmatory diagnosis and flow cytometry, we admitted our patient as an emergency case for complete excision of his mass. Image-guided left medial maxillectomy and left ethmoidectomy were performed overnight under general anesthesia. Upon visualization, the mass had the appearance of a large polyp pedicled to the middle turbinate root, extending laterally to the medial maxilla and superiorly to the ethmoid air cells. It was successfully cleared from the lateral nasal wall and the anterior face of the sphenoid sinus.

**Figure 2:**
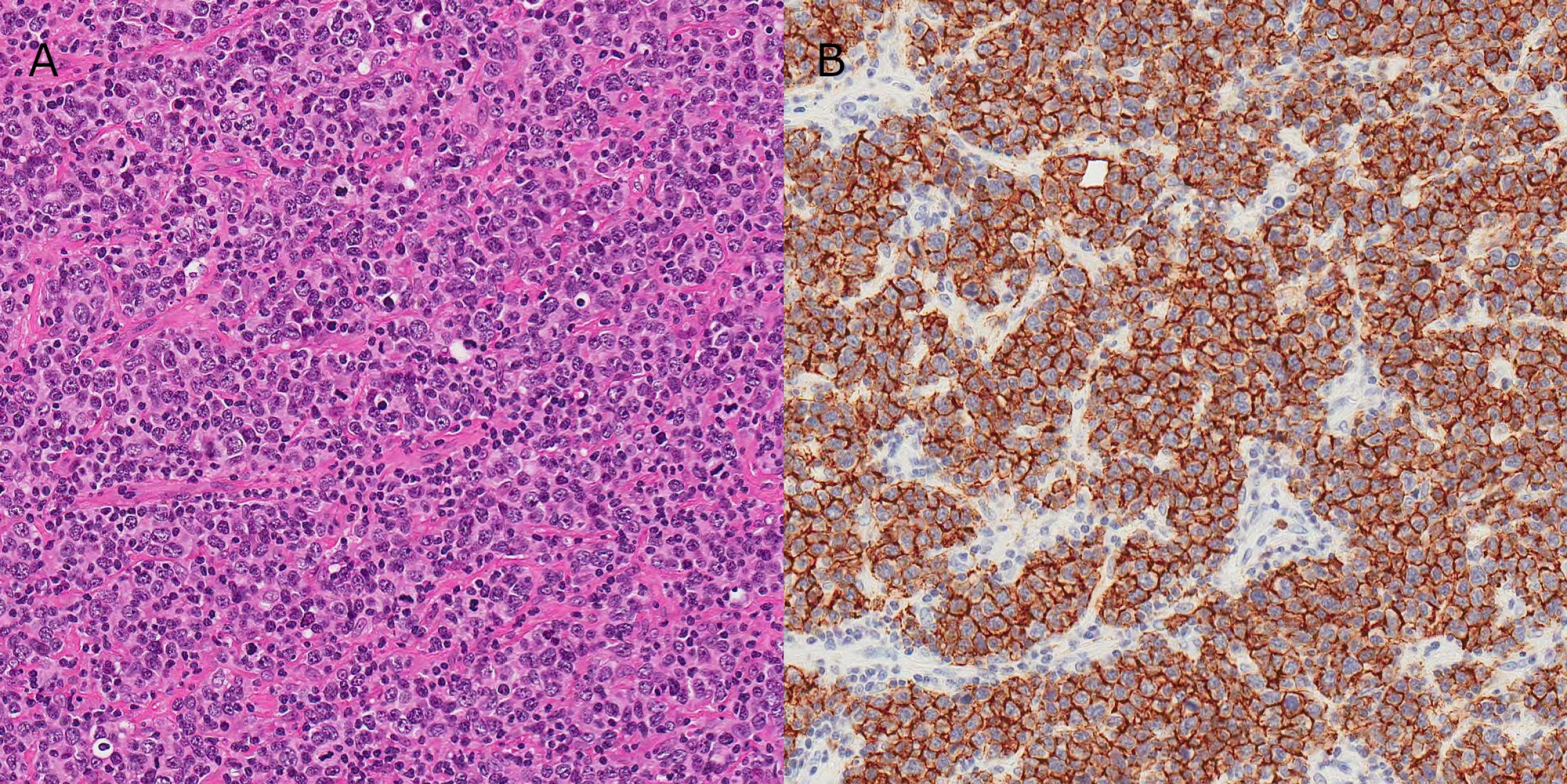
Nasal biopsy specimen under 20X objective with A) hematoxylin and eosin staining and B) CD20 staining showing diffuse proliferation of atypical B lymphocytes.

Immunohistochemistry of the malignant cells was diffusely and strongly positive for CD10, CD20, CD45, Bcl2, and Bcl6, focally positive for MUM1 and CD5, and negative for cytokeratin AE1/AE3, cyclin D1, S100, and CD3 (Figure 2). Flow cytometry analysis of the monoclonal B cell population representing 5% of the total leukocyte count was positive for dim CD19, dim CD5, CD10, CD20, and kappa light chain with 66% viability in 99% of lymph nodes. These findings confirmed the diagnosis of a DLBCL with germinal cell phenotype.

Following the diagnosis, our patient was referred to our hematology colleagues for further investigations and treatment planning. They reviewed the latest CT with the radiologist to determine the extent of the lymphoma. No solid mass was seen in the optic nerve and orbit, and no signs suggesting central nervous system involvement were seen. A lumbar puncture and positron emission tomography (PET) scan were ordered to better understand the disease progression. The former returned normal, but the latter showed high-grade 18F-fluorodeoxyglucose (FDG) uptake in the right ethmoid sinus extending into the right medial rectus muscle and optic nerve. Treatment was initiated with R-mini-CHOP (rituximab and reduced dose of cyclophosphamide, doxorubicin, vincristine, and prednisone) and filgrastim over six three-week cycles for a total of 18 weeks. PET studies following treatment showed resolution of the left-sided mass and mild improvement in the orbital abnormalities. Our patient’s symptoms of nasal congestion, proptosis, headaches, and cranial neuropathies continued to improve, and he was able to complete the chemotherapy cycles without major complications. He remained in remission before being lost to follow-up two months post-treatment.

## Discussion

Malignant lymphomas in the nasal cavity and paranasal sinuses are frequently misdiagnosed based on clinical judgment at a rate exceeding 20% [9]. When they present with the classic nasal presentations, the symptoms have poor specificity that can be confused with infectious or inflammatory pathologies [10]. Nonrhinological symptoms may further complicate or delay the diagnosis. For example, many of these cases may initially be assessed by an ophthalmologist as, once the disease is involving the orbit itself, the patient may be most debilitated by symptoms arising from the orbital or periorbital structures [8]. In such instances, it is of utmost importance that one recognizes the true origin of the pathology, so that appropriate treatment can be initiated in a timely manner. Delayed diagnosis of DLBCL can lead to progression of the disease and debilitating, or even fatal, consequences [10]. Fortunately, our patient received the appropriate investigations following an initial misdiagnosis and achieved remission with emergent treatments.

We present a literature review of proptosis secondary to sinonasal masses in Table 1 [11-16]. To the best of our knowledge, the present case report is the first documented instance of a sinonasal mass causing ocular symptoms exclusively on the contralateral side. Unfortunately, a limitation of our case report is that we cannot definitively establish a cause-effect relationship based on the investigations completed. Notably, our patient’s past MRI scan had poor visualization of the orbit due to motion artifacts, and no biopsy of the orbit was performed. However, we can reasonably draw an association between our patient’s sinonasal DLBCL and his left-sided symptoms, as both resolved with the initiation of chemotherapy.

**Table 1:** Literature review of proptosis caused by masses of the nasal cavity and paranasal sinuses. Six other cases in the current literature of proptosis associated with masses in the nasal cavity and paranasal sinuses are presented. None of these patients had proptosis exclusively on the side contralateral to the mass. DLBCL, diffuse large B-cell lymphoma; HTLV-1, human T-lymphotropic virus type 1; NK, natural killer; ALL, acute lymphoblastic leukemia

Study	Age and sex	Primary site	Other structures involved	Cancer type	Side of proptosis	Chemo-radiation regimen	Outcome
Suzuki et al. [11]	39M	Left ethmoid sinus	Left maxillary sinus, orbit, skull base	Myeloid sarcoma	Left	Daunorubicin, cytarabine, radiotherapy	Remission
Dalmia et al. [12]	50M	Left nasal cavity	Left orbit, cribiform plate, right nasal cavity	DLBCL	Left	Cyclophosphamide, vincristine, doxorubicin, dexamethasone, rituximab	Remission
Yang et al. [13]	42F	Right ethmoid sinus	Right nasal cavity, skull base, orbit	DLBCL	Right	Methotrexate, cytarabine, ifosfamide, vincristine, cyclophosphamide, radiotherapy	Remission
Laveaux et al. [14]	48F	Bilateral nasal cavity, ethmoid sinuses	Bilateral orbits, left optic nerve	HTLV-1 lymphoma	Bilateral	Etoposide, prednisone, vincristine, cyclophosphamide, doxorubicin, rituximab	Remission
Chen et al. [15]	53M	Right ethmoid, sphenoid, frontal sinus	Right orbit	NK/T-cell lymphoma	Right	Cytarabine, doxorubicin, vincristine, cyclophosphamide, allopurinol	Dead of disease
Chang et al. [16]	24M	Right maxillary sinus	Right orbit, nasal vault	ALL	Right	Vincristine, 6-mercaptopurine, etoposide, daunorubicin, cyclophosphamide, cytarabine, indarubicin, mitoxantrone	Dead of pneumonia, sepsis

We propose several theories for how a mass on the left side could conceivably drive edema in the right orbit. First, the FDG uptake seen on PET imaging suggests peritumoral inflammation or direct tumor infiltration into the orbit through the ethmoid sinus [17]. Without a definitive biopsy of the orbit, it is unclear which process is at play. Although not seen on our patient’s imaging studies, involvement of the cavernous sinus would also account for the symptoms consistent with trigeminal and abducens nerve palsies seen in our patient [18]. Given that ethmoid sinusitis commonly precipitates cavernous sinus thrombosis [19], it is conceivable that tumor or inflammatory cells in the ethmoid sinus could spread along the same route. An alternative theory is paraneoplastic syndrome, as documented in a similar case by Harris et al. [20]. Their patient presented with bilateral orbital myositis and multiple neuropathies involving cranial nerves V, VI, and VII, and was diagnosed with a nodal B-cell lymphoma. Based on findings from an EOM biopsy and other investigations, the authors speculated that a paraneoplastic effect of lymphoma was responsible for the collection of symptoms.

## Conclusions

This case highlights an unusual presentation of sinonasal DLBCL that presented a diagnostic challenge. Our patient’s sinonasal pathology was initially ruled out as a possible cause of his contralateral ocular symptoms. Fortunately, our early investigations led to the correct diagnosis of DLBCL when it was not suspected by clinical intuition. To be thorough, it is important to consider malignant processes when there is not an obvious link between a patient’s pathology and symptomatology.
